# Effects of Liquid-to-Solid Ratio, Temperature, and Alkali Activator Concentration on Rheological Properties of Ternary Solid Waste Geopolymer

**DOI:** 10.3390/ma19132923

**Published:** 2026-07-07

**Authors:** Liuyun Huang, Yingjie Zuo, Jiaquan Wang, Yuliang Chen, Tun Li

**Affiliations:** School of Civil Engineering and Architecture, Guangxi University of Science and Technology, Liuzhou 545006, China; 100000635@gxust.edu.cn (L.H.); wjquan1999@163.com (J.W.); ylchen@gxust.edu.cn (Y.C.); 100000644@gxust.edu.cn (T.L.)

**Keywords:** geopolymer grouting material, rheological properties, liquid-to-solid ratio, temperature, alkali activator concentration

## Abstract

To investigate the rheological properties of geopolymer grouting materials, a systematic study was conducted on a slag–red mud–fly ash ternary solid waste geopolymer grouting material (TSWGGM). The effects of liquid-to-solid ratio (0.5–1.5), slurry temperature (10–50 °C), and alkali activator concentration (1.0–1.8 mol/L) on the rheological model, yield stress, time-dependent viscosity behavior, and thixotropy were examined. The results show that the liquid-to-solid ratio is the dominant factor determining the rheological model. With increasing liquid-to-solid ratio, the slurry exhibits Herschel–Bulkley (shear thinning), Bingham, and Newtonian fluid behaviors in sequence. The yield stress decreases significantly with increasing liquid-to-solid ratio and approaches zero at high liquid-to-solid ratios (≥1.0), while it increases with rising temperature. In the temperature range of 20–30 °C, the time-dependent viscosity curves follow an exponential growth law, whereas at 40–50 °C, competition between early reaction and shear destruction leads to an initial decrease followed by an increase in viscosity. At low alkali activator concentrations (≤1.4 mol/L), the time-dependent viscosity curves still obey the exponential model, but at excessively high concentrations (≥1.6 mol/L) a non-monotonic change (an initial increase followed by a decrease, and final steady growth) occurs. The thixotropic loop area decreases with increasing liquid-to-solid ratio, first decreases and then increases with rising temperature, and exhibits a critical phenomenon of first increasing and then decreasing with increasing alkali activator concentration.

## 1. Introduction

China has clearly defined its “dual carbon” goals. However, the construction of transportation infrastructure yields a substantial carbon footprint, primarily driven by the massive consumption of carbon-heavy materials (particularly Portland cement, steel, and asphalt) and fossil fuel combustion during material transport and equipment operations. Portland cement production alone is responsible for approximately 7–8% of global anthropogenic CO_2_ emissions [1]. Therefore, utilizing green building materials to reduce carbon emissions from the source represents a critical pathway to fulfill this national commitment, which targets peaking carbon dioxide emissions before 2030 and achieving carbon neutrality before 2060. Geopolymer grouting materials are an outstanding type of green building material in the field of engineering restoration. They simultaneously meet the requirements of environmental protection and performance, and thus have been widely promoted.

The rheological properties of grouting slurries (primarily cement- and geopolymer-based slurries) have a significant impact on the migration and diffusion of grouting materials, serving as a prerequisite for studying their migration and diffusion models. They also provide the basis for guiding the rational design of cement slurry water–cement ratio, grouting pressure, time, and grouting process in on-site grouting, and have a substantial influence on slurry diffusion and migration [2,3]. Single-precursor or binary solid waste geopolymer systems possess distinct engineering bottlenecks. Single-precursor granulated blast furnace slag (GGBS) alkali-activated binders usually exhibit rapid setting and severe autogenous or drying shrinkage, which induces microcracking [4,5]. Binary fly ash–red mud (FA-RM) composites, although more cost-effective, typically yield inadequate unconfined compressive strength (frequently ≤3.1 MPa at 28 days) due to the low early-age reactivity of Bayer red mud and Class F fly ash in the absence of an active calcium source [6]. To overcome these limitations, researchers have turned to ternary solid waste composite systems. Li et al. [7] synthesized a ternary cementless fly ash-based geopolymer incorporating red mud and blast furnace slag, systematically evaluated its fluidity, strength, and anti-permeability, and revealed that slag incorporation promoted the conversion of N-A-S-H to C-A-S-H gel, thereby mitigating the mild strength degradation caused by high red mud dosages. Wang et al. [8] evaluated the mechanical properties, workability, and cost-effectiveness of ternary solid waste geopolymers prepared by incorporating red mud into a slag–fly ash system, and demonstrated that an optimal red mud substitution rate of 20 wt% effectively stimulated the depolymerization of aluminosilicate precursors, yielding a balanced initial fluidity of 254 mm and a 28-day compressive strength of 34.2 MPa.

The rheological properties of the slurry of conventional cement-based grouting materials are related to multiple factors such as the water–cement ratio and temperature [9]. Shao et al. [10] found that as the temperature rises, the shear stress and rheological index of cement-based composite slurry increase, while the consistency coefficient decreases. Yang et al. [11] found that the higher the water–cement ratio, the lower the viscosity of the slurry, and the better the fluidity, but the slurry was less stable and more prone to segregation. Since the influence of alkaline activators in the slurry of geopolymer grouting materials cannot be ignored [12], when studying the rheological properties of geopolymer grouting materials, in addition to considering conventional factors such as liquid–solid ratio and temperature, the impact of alkaline activators should also be discussed [13,14].

When conducting research on rheological properties, the research content mainly focuses on three core parts. The first is the rheological model and key parameters [15,16]. It is determined that the rheological model is the cornerstone of the research, aiming to describe the flow behavior of the slurry with a mathematical model and quantify its core characteristics. Under the selected model, the minimum shear stress required for the slurry to start flowing is determined. That is, the yield stress is measured. The second is the time-varying viscosity [17,18], which is the most notable feature that distinguishes grouting materials from ordinary fluids. The viscosity of the slurry is not constant but continuously changes from the start of stirring to the final solidification, which is of great significance for establishing the diffusion model. The third is thixotropy. Ye et al. [19] found that the longer the standing time, the higher the temperature, and the larger the solid volume fraction, the more difficult the structural failure and the subsequent structural reconstruction process become, indicating that the reversibility of thixotropic behavior is lower. The slurry becomes thin and easy to flow in the high-shear environment of pumping and pipeline transportation. Once injected into the stratum and at rest, the viscosity rises, and strength can be quickly established to prevent loss.

Traditional cementitious suspensions are primarily composed of irregular, sharp-edged angular cement particles and water. During the initial stage of hydration, their rheological parameters are predominantly governed by physical effects, where particles assemble into a reversible flocculation structure driven by Van der Waals forces, dominating the thixotropy of the paste and imparting its initial static yield stress [20]. As the reaction progresses, chemical effects become dominant, whereby the in situ-generated C-S-H gel network gradually transforms the loose physical flocculation skeleton into an irreversible rigid structure, leading to a continuous growth in viscosity over time [21]. In contrast, geopolymer grouting materials are composed of multicomponent precursors and alkali activators. From a physical perspective, the complex geometric heterogeneous morphology of the precursors, combined with the initial viscosity of the activator solution that is significantly higher than that of water, endows the geopolymer with a distinct initial rheological response. From a chemical perspective, the network formation driven by the “dissolution–polycondensation” process, induced by the highly reactive activator, becomes dominant at an earlier and more aggressive stage, giving rise to a unique three-dimensional amorphous gel structure [22,23]. Given the fundamental differences between the two systems regarding their solid-phase matrix, the nature of the liquid phase, and the kinetics of microstructural network formation, a thorough investigation into the early-age rheological properties of geopolymer grouting materials holds significant academic and engineering value.

For the reasons mentioned above, in this paper, rheological research is expanded from the traditional cement-based system to solid waste geopolymer grouting materials, supplementing the study of the rheological properties of geopolymer grouting slurry. The aim is to provide theoretical support and data for the optimization of the proportion of geopolymer grouting materials, the determination of pumping process parameters, and the evaluation of injectability in engineering practice. In the experiment, the effects of the liquid–solid ratio, temperature, and the concentration of alkali activator on the rheological properties of the slurry were investigated by preparing a ternary solid waste geopolymer grouting material (TSWGGM) of slag–red mud–fly ash.

## 2. Materials and Methods

### 2.1. Raw Materials

The red mud used is sintered red mud, and the alkali activator uses instant sodium silicate with a modulus of 1, which is provided by Henan Borun Foundry Materials Co., Ltd. (Zhengzhou, China). The slag micro-powder is of S95 grade, and the fly ash is of Class F, provided by Gongyi Longze Water Purification Materials Co., Ltd. (Zhengzhou, China). The main chemical components of red mud, slag, and fly ash were analyzed using a Shimadzu EDX-7200 energy dispersive X-ray fluorescence spectrometer (XRF) (Shimadzu Corporation, Kyoto, Japan), and the results are shown in Table 1. It can be seen that the precursors (red mud, fly ash, and slag) mostly exist in the form of oxides formed by elements such as Al, Si, Ca, Fe, Mn, and S. Sintered red mud is characterized by a high calcium content [24], which can provide part of the calcium source, promote the formation of C-A-S-H in synergy with slag, and endow the system with strong alkaline characteristics as a supplement to the alkaline activator. Slag serves as the main calcium source and an active silicon and aluminum source for the formation of C-A-S-H. Due to its low calcium content [25], F-class fly ash, when used as a precursor, provides additional Si^4+^ and Al^3+^. This Al^3+^ will enter the slag-dominated C-A-S-H network, reducing the Ca/Si ratio of the gel and increasing its Al/Si ratio, which can form a denser and more stable C-(N)-A-S-H hybrid gel.

**Figure 1:**
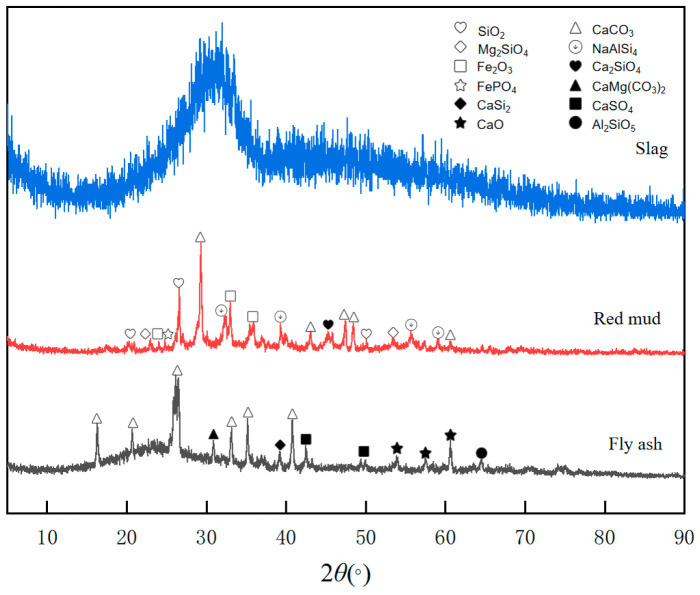
XRD patterns of red mud, slag, and fly ash.

**Figure 2:**
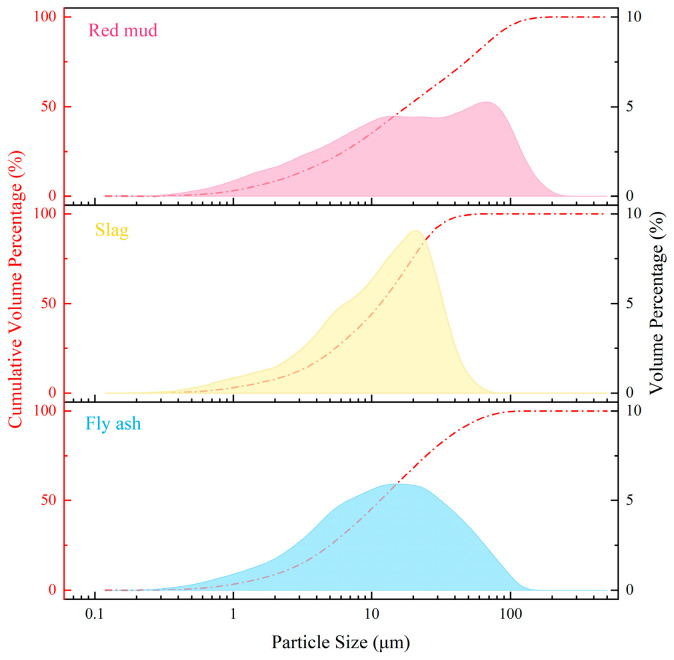
Particle size distribution map of materials.

**Figure 3:**
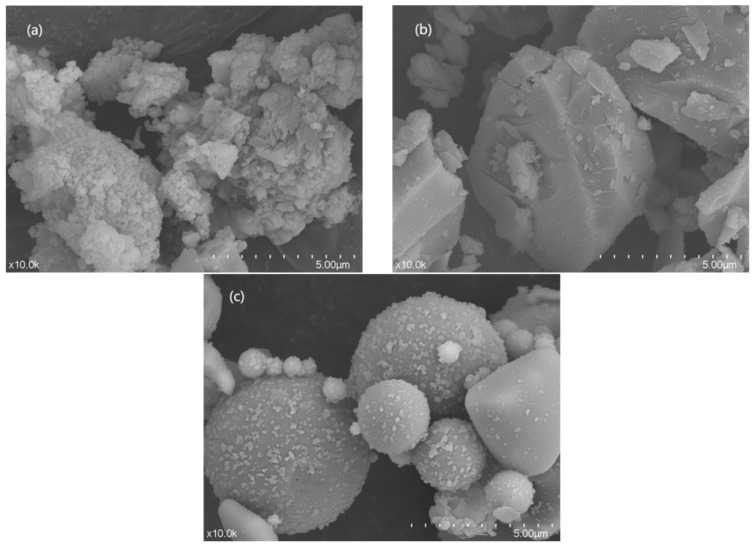
SEM images of materials: (**a**) red mud; (**b**) slag; (**c**) fly ash.

The phase composition was analyzed using a Bruker D8 ADVANCE X-ray diffractometer (XRD) (Bruker AXS SE, Karlsruhe, Germany), and the results are shown in Figure 1. The main crystal phases of fly ash are high-calcium minerals and aluminum- and silicon-containing minerals, while red mud is mainly composed of high-aluminum, high-iron, and high-silicon minerals. Both have relatively sharp diffraction peaks and good crystallinity. In contrast, the XRD pattern of the slag has a higher background, presenting a distinct “steamed bun peak” feature with fewer sharp diffraction peaks, indicating that it contains a large amount of amorphous phase or has poor crystallinity. This structural feature is conducive to the chemical bond breakage of the slag during the hydration process, thereby providing higher gelling activity. The particle size distribution and microscopic morphology of red mud, slag, and fly ash were analyzed by a Fubbs FBS-1076Z laser particle size (Fubbs, Jinan, China) and German Zeiss Gemini Sigma 300 VP SEM field emission scanning electron microscope (SEM) (Carl Zeiss AG, Oberkochen, Germany), as shown in Figure 2 and Figure 3. The average particle size (D50) of red mud, fly ash, and slag particles is 17.95 μm, 11.54 μm, and 11.62 μm, respectively.

Scanning electron microscopy (SEM) observations indicated that red mud, slag, and fly ash presented significantly different microscopic morphologies. Red mud is a complex and heterogeneous aggregate with a rough and porous surface. A rough surface increases the mechanical frictional resistance between particles. On the one hand, the porous structure increases the specific surface area and promotes the degree of hydration reaction; on the other hand, it adsorbs free water and reduces the effective water–cement ratio. Both of these factors jointly lead to the thickening of the slurry.

Slag is in the form of angular blocky or polygonal fragments, with a relatively smooth and dense surface and a glassy luster. Although the smooth surface reduces the coefficient of friction, the angular shape still causes mechanical meshing between particles, increasing the flow resistance of the slurry.

Fly ash is mainly composed of regular spherical particles, with a smooth surface. Some microspheres have smaller particles adhering to their surfaces. Spherical particles can freely roll in the slurry, converting sliding friction into rolling friction, thereby significantly reducing internal frictional resistance and improving the fluidity of the slurry.

### 2.2. Sample Preparation

To explore the effects of temperature, liquid–solid ratio, and alkali activator on the rheological properties of the ternary solid waste geopolymer grouting material of slag–red mud–fly ash, in this study, the grouting material was prepared with a mass ratio of slag, red mud, and fly ash of 3:1:1 based on a large number of previous experiments [8,26]. The experimental scheme design is shown in Table 2.

Firstly, according to the experimental design, weigh the corresponding mass of raw materials and pre-mix them using an electric mixer to obtain uniform powder. Meanwhile, the alkali activator solution should be prepared by dissolving instant sodium silicate powder in laboratory-grade purified water (commercially sourced Wahaha purified water, Hangzhou, China, featuring an electrical conductivity of 1.56 μS/cm) to achieve the target concentration. Turn on the water bath constant-temperature box to preheat the slurry material. After the water bath temperature reaches the preset value and remains constant for 1 h, mix the raw material powder with the sodium silicate solution and stir it using a vertical mixer. Within 15 s after the stirring is completed, transfer the slurry to the sample cup, assemble, and connect it to the rotational rheometer.

### 2.3. Test Method

**Figure 4:**
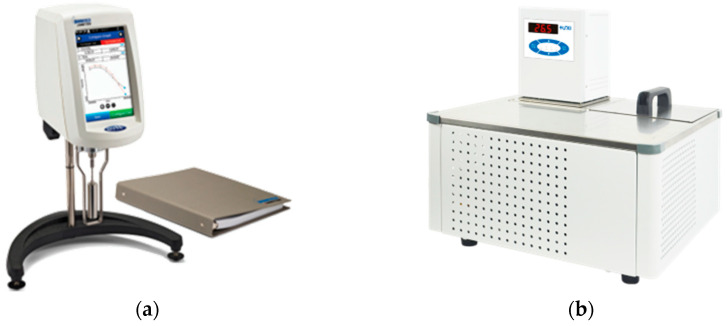
Experimental instrument: (**a**) rotational rheometer; (**b**) horizontal high- and low-temperature constant-temperature bath.

The test instruments are shown in Figure 4. The rheological tests and time-varying viscosity tests were conducted using the Brookfield DVNext rotational rheometer from the United States (AMETEK Brookfield, Middleboro, MA, USA). The slurry temperature was controlled by the HLC-2008SHW horizontal high- and low-temperature constant-temperature bath (Shanghai Huxi Industrial Co., Ltd., Shanghai, China), with a temperature control range of −20 °C to 100 °C and an accuracy of ±0.1 °C.

The rotational speed is selected from the range coefficient formula in the DVNext operation manual. The range coefficient = rotational speed × viscosity.

The rotor used in this test is SC4-21, with a range coefficient of 50,000. Rheological measurements were performed using a coaxial cylinder geometry (Searle-type, consisting of a stationary outer cup and a rotating inner bob) equipped with a Small Sample Adapter (SSA). An SC4-21 spindle with a range coefficient of 50,000 was employed for all tests. During the experiment, the rotational speed (RPM) and torque were recorded in real time. These physical quantities were subsequently converted into rheological parameters; specifically, the shear stress and shear rate were automatically calculated via the Rheocalc T 2.1.53 software based on the narrow-gap solution of the Margules equation, utilizing the built-in geometric factors (Spring Torque Scale and spindle constants).

The plastic viscosity of the TSWGGM slurry measured in the pre-experiment was between 250 and 300 cP. During the calculation, 500 cP was taken, and the calculation speed was 100 r/min. The shear rate was calculated as 93 s^−1^ based on the rotor size.

#### 2.3.1. Sample Loading and Pre-Conditioning

Upon completion of mixing, the geopolymer slurry was transferred into the sample cup of a coaxial cylinder rheometer (equipped with a Small Sample Adapter, SSA) within 30 s. The loading process was performed in steps to prevent slurry overflow and bubble entrapment: approximately one-third of the cup volume was initially filled before inserting the SC4-21 spindle, followed by supplementing the remaining slurry up to the volume indicator line. After securing the sample cup, tightening the spindle, and connecting the temperature probe, the testing program was initiated immediately. To eliminate the mechanical shear history accumulated during loading and to ensure thermal equilibrium, all samples underwent an identical pre-conditioning procedure prior to formal loading. The sample was first subjected to continuous pre-shearing at a constant shear rate of 93 s^−1^ for 30 s to disrupt any random, initial networks, thereby ensuring that all specimens reached a consistent “structureless” or “fully dispersed” reference state. Subsequently, a 30 s rest period was maintained to allow for partial microstructural recovery and the relaxation of residual stresses.

#### 2.3.2. Rheological Curve Measurement

Immediately following the pre-conditioning sequence, a stepped loading procedure in controlled shear rate mode was executed to obtain the rheological curves. Up-ramp stage: The shear rate was linearly increased from 0 s^−1^ to 93 s^−1^ in a stepwise manner over 1 min. Down-ramp stage: Subsequently, the shear rate was decreased from 93 s^−1^ back to 0 s^−1^ over another 1 min. The system automatically recorded the shear stress and shear rate data during both the up- and down-ramp stages, which were subsequently utilized to evaluate the yield stress, thixotropy, and rheological model fitting of the geopolymer grouting materials.

Specifically, two distinct types of yield stress were evaluated to describe the structural and flowing properties of the geopolymer slurry. The static yield stress (τ_s_) was determined directly from the experimental up-ramp flow curve, defined as the initial shear stress captured at the first measurement point of the rheometer during the rotor startup, reflecting the critical stress required to transition the stagnant slurry into macroscopic flow. In contrast, the dynamic yield stress (τ_0_) was mathematically derived by fitting the experimental flow curves obtained from the steady-state down-ramp stage with appropriate fluid models to characterize the flow behavior after complete structural breakdown.

#### 2.3.3. Time-Dependent Viscosity Test

Following sample loading and pre-conditioning procedures (pre-shearing at 93 s^−1^ for 30 s, followed by a 30 s rest period), the instrument was controlled to apply a constant shear rate of 93 s^−1^ for a continuous duration of 1 h. The apparent viscosity data as a function of time were collected in real time throughout the shearing process.

Rheological models abstract complex material behavior into concise mathematical equations by establishing the relationship between variables such as shear stress and shear rate.

The rheological models used in this paper are as follows.

The Herschel–Bulkley rheological model has the following rheological equation:


(1) $$\tau =\tau_{0}+K\gamma^{n}$$

In the formula, *τ*_0_ represents the dynamic yield stress (Pa); K is the consistency coefficient (Pa·sn); n is the rheological index with a dimension of 1; *γ* is the shear rate (1/s).

The Bingham rheological model has the following rheological equation:


(2) $$\tau =\tau_{0}+\mu_{P}\gamma$$

In the formula, *μ_p_* represents plastic viscosity (Pa·s).

The Newtonian rheological model has the following rheological equation:


(3) τ=μγ

In the formula, *μ* represents dynamic viscosity (Pa·s).

## 3. Results and Discussion

### 3.1. Influence of Liquid–Solid Ratio on Rheological Properties

**Figure 5:**
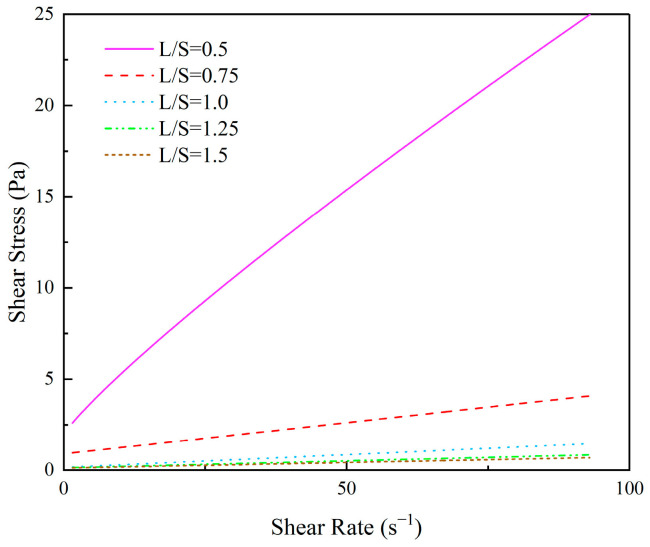
Shear stress versus shear rate curves of slurries under different liquid-to-solid ratio.

Figure 5 shows the shear stress and shear rate curves of the slurry under different liquid–solid ratios, and Table 3 presents the rheological equations for fitting TSWGGM slurries with different liquid–solid ratios. When the liquid–solid ratio is 0.5, the slurry conforms to the H-B (Herschel–Bulkley) rheological model, with a rheological index n less than 1, showing shear-thinning fluid. When the liquid–solid ratio is 0.75, it conforms to the Bingham rheological model. When the liquid–solid ratio is 1.0, 1.25, and 1.5, it conforms to the Newtonian rheological model. The test results show that with the increase in the liquid–solid ratio, the rheological model of the TSWGGM slurry changes from H-B-type to Bingham-type and then to Newtonian-type. At a higher liquid–solid ratio, the particle concentration was sufficiently diluted, causing the distance between the particles to exceed the range of colloid attraction. As a result, mechanical connections and the friction between the particles were minimized, and the macroscopic flow was mainly dominated by the fluid dynamic viscosity dissipation in the continuous liquid phase. Due to the inherent Newtonian fluid property of the alkaline sodium silicate activator solution with high viscosity, the highly diluted ternary suspension exhibited characteristics close to those of a Newtonian fluid. Furthermore, at the same shear rate, the shear stress of the slurry gradually increases as the liquid–solid ratio decreases.

Static yield stress (τ_s_) is defined as the critical minimum shear stress required to initiate material flow. Below τ_s_, the material exhibits elastic solid-like behavior, maintaining its initial shape and resisting permanent deformation; once the applied shear stress exceeds τ_s_, the internal network yields, and the material begins to flow like a fluid.

Physically, τ_s_ is experimentally determined under quiescent conditions, representing the peak stress required to rupture microstructural physical flocs and early-stage chemical gel linkages. Conversely, dynamic yield stress (τ_0_) is calculated by fitting the steady-state down-ramp rheological curve using the Herschel–Bulkley (Equation (1)) or Bingham (Equation (2)) models, describing the minimum stress required to maintain flow after the structural network has been fully destroyed by shear. Due to the rapid formation of early-stage geopolymerization gel networks during the rest period, the static yield stress of the geopolymer slurry is prominently higher than its dynamic counterpart.

**Figure 6:**
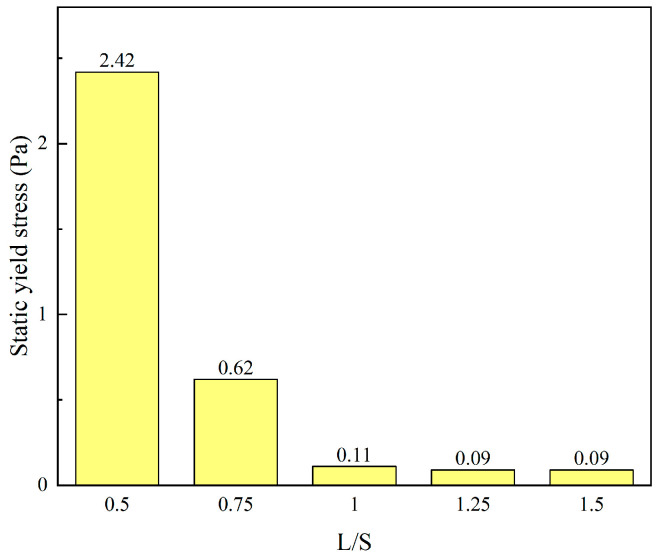
Static yield stress under different liquid-to-solid ratios.

Figure 6 illustrates the evolution of the static yield stress of the slurries at various liquid-to-solid (L/S) ratios. It is observed that τ_s_ exhibits a significant declining trend with the increase in the L/S ratio. When the L/S ratio exceeds 1.0, τ_s_ approaches zero asymptotically.

This phenomenon indicates that within high L/S ratio systems, the initial microstructural network of the slurry is highly susceptible to shear degradation, rendering its early-stage rheological behavior close to that of a Newtonian fluid with a negligible yield stress. Under such conditions, the slurry exhibits superior fluidity, injectability, and pumpability, which highly facilitates its sufficient permeation and dispersion within geomedia, thereby effectively lowering the initial pumping pressure and reducing the energy consumption load of pumping equipment.

The time-varying viscosity refers to the property that the viscosity of the slurry continuously develops with the increase in hydration time [27]. A large number of studies have shown that the viscosity of the slurry is always in a dynamic state during the grouting diffusion process [28]. If the time-varying viscosity is not taken into account in the study of slurry diffusion models, the calculated diffusion radius will be too large, resulting in a significant deviation between the theoretical results and the actual situation [29,30].

**Figure 7:**
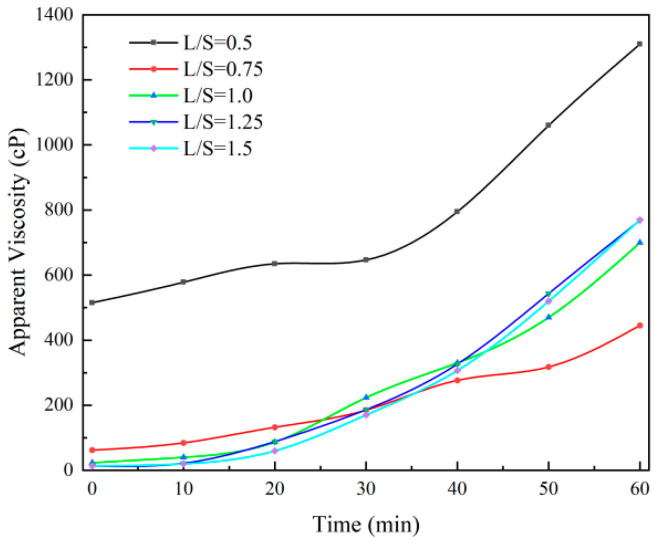
Time-dependent viscosity curves under different liquid-to-solid ratios.

Figure 7 shows the time-varying viscosity curves under different liquid–solid ratios, and Table 4 presents the time-varying viscosity equations of the slurry under different liquid–solid ratios. It can be seen that the viscosity of the slurry shows obvious time-varying characteristics over time. The exponential time-varying model has higher numerical convergence and fitting robustness when describing the early fluid retention period and the flow resistance of the slurry before initial setting. Its variation law can be represented by a unified exponential function [31]:


(4) $$\mu \left(t\right)=\delta e^{\beta t}$$

In the formula, *δ* represents the initial viscosity (cP) of the slurry; *β* is the time-varying coefficient of viscosity; t represents the grouting time (s); *μ*(*t*) is the viscosity (cP) at time t.

It can be seen from the figure that the initial viscosity decreases as the liquid–solid ratio increases. An increase in the liquid–solid ratio indicates an increase in the mass fraction of the liquid phase and a decrease in the mass fraction of the solid phase. At the initial moment before the reaction is initiated, the initial viscosity of the slurry is already lower, and both the apparent viscosity and yield stress decrease with the increase in the L/S ratio. This sets a lower starting point for the subsequent time-varying process.

The final viscosity of the slurry with a liquid–solid ratio higher than 0.5 at the same time is much lower than that of the slurry with a liquid–solid ratio of 0.5, indicating that a high liquid–solid ratio system can make the time window for maintaining the pumpable state longer.

The thixotropy of the slurry can be expressed by the size of the thixotropic loop area (the difference between the integral of the upward curve and the downward curve) [32]. The area of the thixotropic loop reflects the storage size of thixotropic energy, that is, the difference between the energy required for the disintegration of the structure in the slurry and the energy required for the formation of the structure.

**Figure 8:**
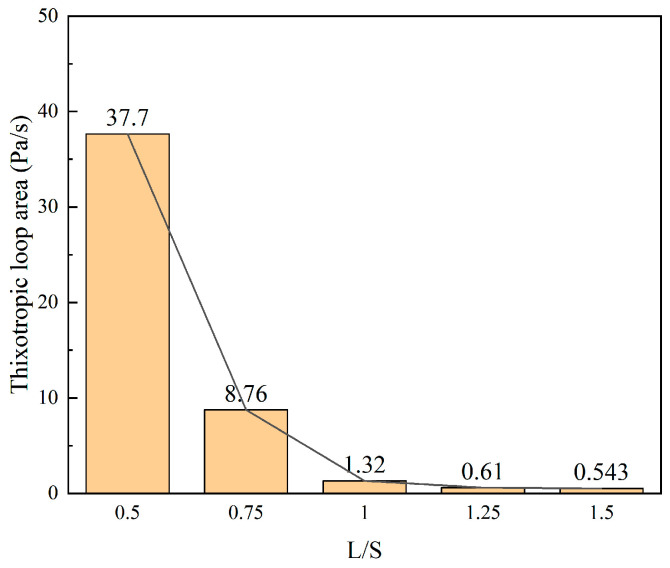
Thixotropic loop area of slurry under different liquid-to-solid ratios.

Figure 8 shows the thixotropic loop area of the slurry under different liquid–solid ratios. It can be seen that as the liquid–solid ratio increases, the thixotropy of the slurry decreases, and the thixotropic loop surface of the slurry with a high liquid–solid ratio is actively lower, approaching zero. This indicates that when the liquid–solid ratio is 0.5 and 0.75, the slurry structure is significantly damaged, and the mechanical energy dissipated due to the destruction of microstructure in a single shear cycle is relatively large. Under high liquid-to-solid ratios, the prominent reduction in the thixotropic hysteresis loop area of the grout is primarily attributed to the physical dilution effect. As the volumetric proportion of the liquid phase increases, the solid volume fraction within the suspension decreases significantly, which elongates the average inter-particle distance and renders the initial physical flocculation network extremely fragile [33]. Consequently, during dynamic shear cycles, the mechanical energy dissipation required to rupture the microstructural network approaches zero. These characteristics of low yield stress and low viscosity impart excellent injectability to high liquid-to-solid ratio grouts, making them uniquely suitable for long-distance, low-resistance pumping as well as penetration grouting into micro-fissures. However, for conventional grouting engineering, an ideal slurry must possess highly controllable, dual thixotropic effects: during the pumping and pipeline transportation stage (high-shear zones), the internal network structure of the grout should be rapidly dismantled under intense shear agitation, exhibiting pronounced shear-thinning behavior to minimize pipeline resistance and pump energy consumption. Conversely, once the grout reaches the designated location within the stratum (such as large cavities or zero/low-shear zones characterized by groundwater ingress), the static structure of the material must undergo rapid reconstruction (structural build-up). This triggers a sharp increase in viscosity within a short timeframe, thereby effectively resisting dynamic water washout, controlling the grout spreading radius, and preventing ineffective grout loss.

The most direct physical consequence of an increase in the liquid–solid ratio is a decrease in the mass fraction of solids. In a suspension system, the intensity of interaction between particles and the connectivity of the network are highly dependent on the concentration of solids. When the L/S ratio increases, more liquid phase dilutes the solid particles, the particle spacing increases, and the connectivity of the condensation network is weakened.

The change in the liquid–solid ratio not only alters the physical dilution degree but also affects the chemical kinetics of the polymerization reaction. When the L/S ratio is relatively low, the solid mass fraction is large; that is, the concentration of the precursor material in the reaction system is high, which promotes the dissolution and polycondensation reaction of the aluminosilicate precursor. A large amount of gel products can be generated in the early stage, and these products will further fill the particle gaps and strengthen the network structure.

### 3.2. Influence of Slurry Temperature on Rheological Properties

**Figure 9:**
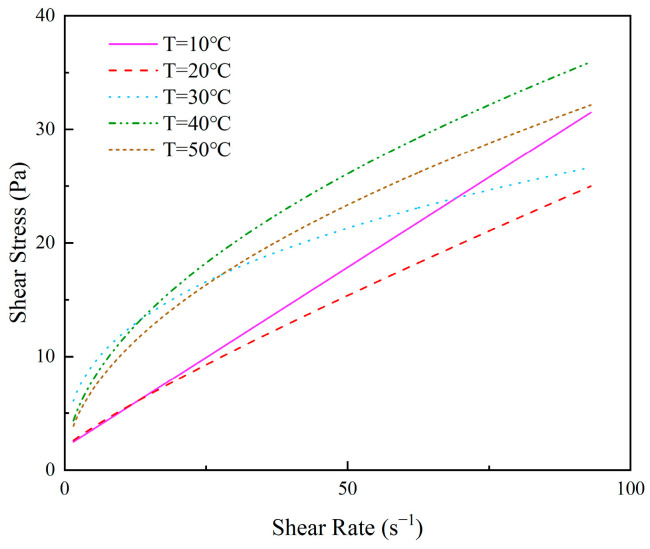
Shear stress versus shear rate curves of slurries under different slurry temperatures.

Figure 9 shows the shear stress and shear rate curves of the slurry at different slurry temperatures, and Table 5 presents the rheological equations of the TSWGGM slurry fitting at different slurry temperatures. When the slurry temperature is 10 °C, the slurry conforms to the Bingham rheological model. When the slurry temperature is 20 °C, 30 °C, 40 °C, and 50 °C, the slurry conforms to the H-B (Herschel–Bulkley) rheological model, with the rheological index n being less than 1, showing shear-thinning fluid.

**Figure 10:**
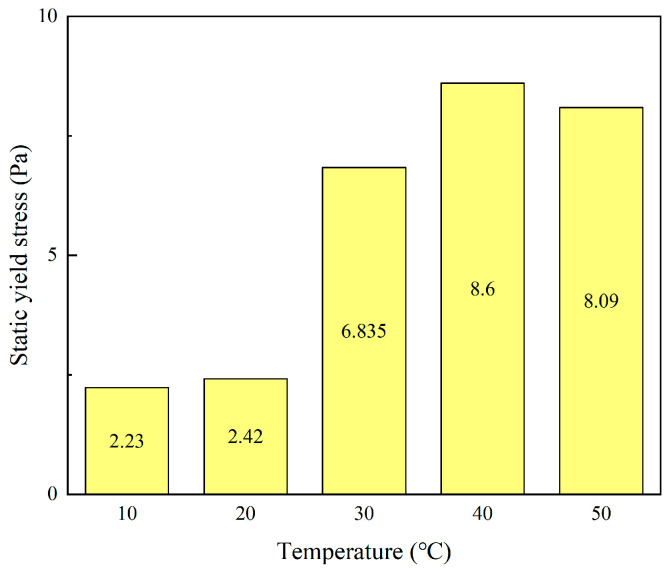
Static yield stress of slurry under different slurry temperatures.

Figure 10 shows the static yield stress of the slurry at different temperatures. It can be seen that the static yield stress of the slurry increases with the rise in temperature, but the error is relatively large under high-temperature conditions, showing a certain degree of instability. The static yield stress at 30 °C increased by 182.4% compared with that at 20 °C. For slurries with hydration or polymerization reactivity, an increase in temperature leads to a growth in the reaction rate [34], which significantly accelerates the hydration of the slurry, and the static yield stress increases with the rise in temperature.

**Figure 11:**
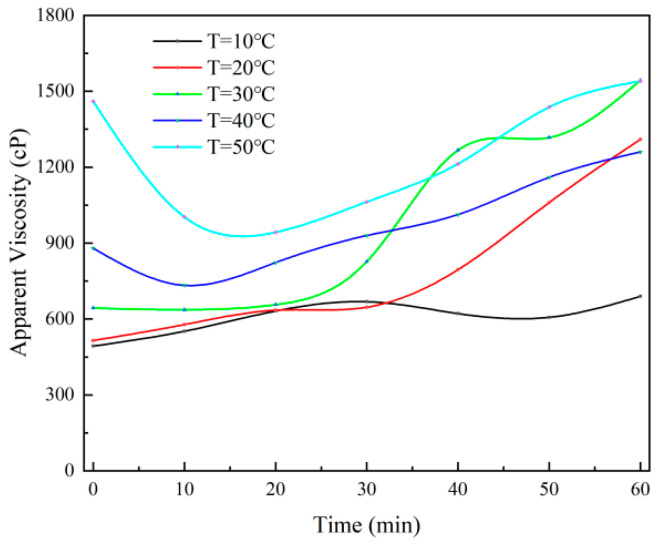
Time-dependent viscosity curves under different slurry temperatures.

Figure 11 shows the time-varying curves of viscosity at different temperatures. It can be observed that the initial viscosity of the slurry is relatively high at 40–50 °C, and then it shows a trend of first decreasing and then increasing, which does not conform to the exponential relationship of formula (4); the time-varying curve of viscosity of the slurry at 20–30 °C still follows the pattern of formula (4), with the viscosity steadily increasing over time, and the viscosity growth of the slurry is slow at 10 °C.

It can be seen from the time-varying curve of slurry viscosity in Figure 11 that the initial viscosity increases with the rise in temperature. The slurry exhibits a pronounced initial viscosity at 40–50 °C. This phenomenon stems not merely from accelerated hydration kinetics and the resultant early gel accumulation [35], but primarily from the temperature-enhanced dissolution of precursors that liberates a vast amount of ions [19,36]. This surge in ion concentration dramatically elevates the ionic strength of the pore solution, severely compressing the electrical double layer on particle surfaces. Consequently, short-range van der Waals attractive forces dominate, driving spontaneous particle agglomeration into a loose, reversible physical flocculation network that macroscopically manifests as a false set [37]. Under continuous shearing by the rheometer rotor, this physical structure is progressively disrupted via mechanical de-flocculation. The release of structural free water previously entrapped within the flocs increases the inter-particle water film thickness and minimizes internal friction, leading to thixotropic shear thinning and a continuous viscosity drop. Upon reaching a minimum viscosity threshold, the reversible physical network is thoroughly dismantled, allowing the thermally activated reaction to assume absolute dominance. The rapid growth of gelation products (C-(N)-A-S-H) at the geometric contact points between particles ultimately drives the subsequent slurry viscosity upturn. The time-varying curve of slurry viscosity shows a slow increase in viscosity when the slurry temperature is 10 °C. This is because the reaction rate of the geopolymer system is significantly suppressed, and the solid phase in the slurry is mainly unreacted precursor particles, with fewer newly formed gel products, which have a relatively low impact on the flow resistance of the slurry. The initial viscosity of the slurry increases with the rise in temperature because the higher the temperature, the more gel products are formed after pre-shearing.

**Figure 12:**
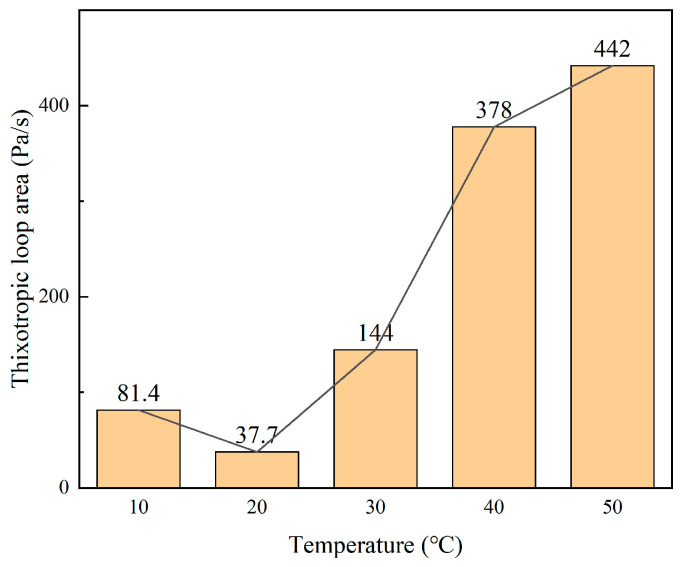
Thixotropic loop area of slurry under different slurry temperatures.

Figure 12 shows the area of the thixotropic loop of the slurry at different temperatures. The area of the thixotropic loop decreases from 81.4 Pa/s to 37.7 Pa/s at 10 °C to 20 °C. From 20 °C to 50 °C, it increases step by step from 37.7 Pa/s to 144 Pa/s to 378 Pa/s to 442 Pa/s.

The reason for this phenomenon may be that the reaction rate of the geopolymer system is relatively slow at 10–20 °C. At this time, the viscosity mainly depends on the liquid phase (alkaline initiator solution), and the viscosity of the liquid will significantly decrease with the increase in temperature [38]. The Brownian motion of the particles intensifies, and the initial structure of the physical lap is more easily broken and recovers more slowly, resulting in a decrease in the area of the thixotropic loop. At temperatures ranging from 20 to 50 °C, the rate of hydration reaction dominates the formation of microstructure. The higher the temperature, the faster the gelation products are generated, leading to an increase in energy consumption for shear failure and reconstruction (i.e., the area of the thixotropic loop).

### 3.3. Influence of Alkali Activator Concentration on Rheological Properties

**Figure 13:**
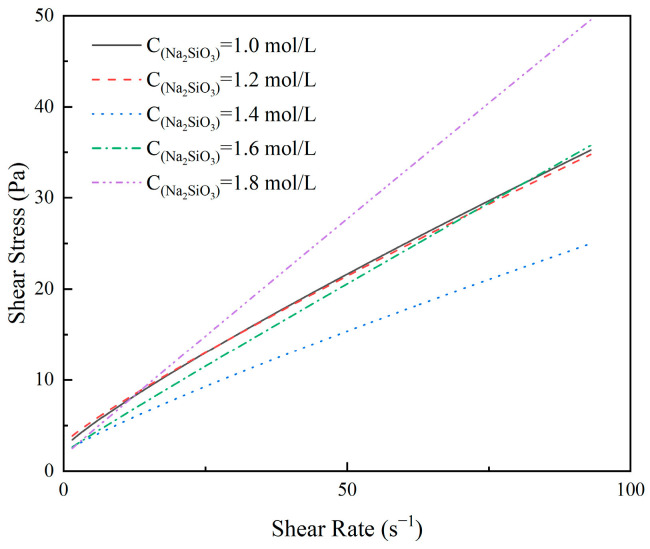
Shear stress versus shear rate curves of slurries under different alkali activator concentrations.

Figure 13 shows the shear stress and shear rate curves of the slurry at different concentrations of alkali activators, and Table 6 presents the rheological equations of the TSWGGM slurry fitted with different concentrations of alkali activators. When the concentration of the alkali activator is 1.0, 1.2, 1.4, and 1.6 mol/L, the slurry exhibits the H-B (Herschel–Bulkley) rheological model, with the rheological index n less than 1, and the slurry is a shear-thinning fluid. When the concentration of the alkali activator is 1.8 mol/L, the slurry conforms to the Bingham rheological model. The rheological index n increases with the increase in the concentration of the alkali activator. When the concentration of the alkali activator is 1.8 mol/L, it can be regarded as a rheological index n of 1. When the concentration of the alkali activator is too high, the rheological index n may be greater than 1, transforming into a shear-thickened fluid, showing swelling fluidity [39,40]. During high-speed pumping, the viscosity will rise sharply, which may cause a sudden surge in pipeline pressure and trigger a pipe burst accident.

**Figure 14:**
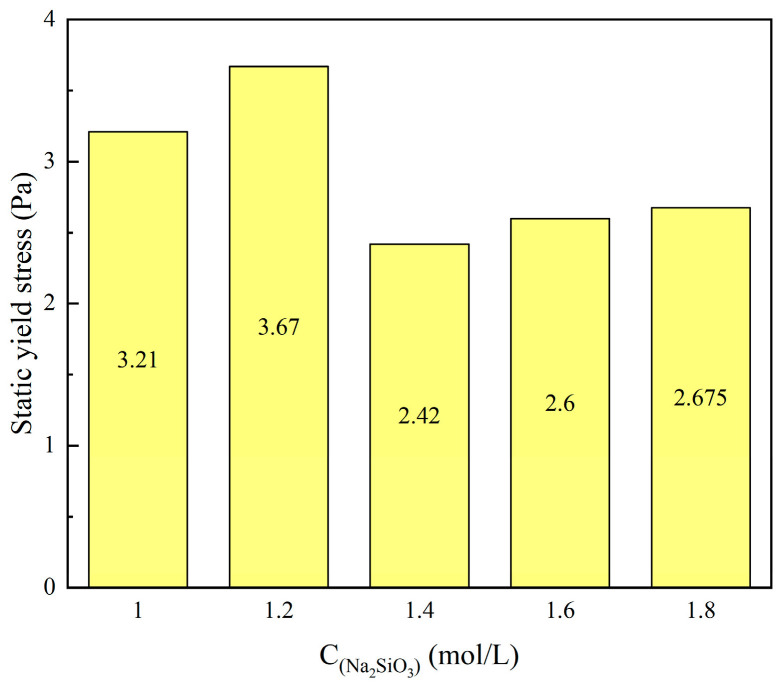
Static yield stress of slurry under different alkali activator concentrations.

Figure 14 shows the static yield stress under different concentrations of alkaline activators. It is worth noting that the variation in the static yield stress τs of the TSWGGM slurry in Figure 14 with the concentration of the activator follows a consistent pattern with the segmented characteristics of the thixotropic loop area (discussed later in this section). This phenomenon can be explained by surface electrochemical theory. According to the research of Kashani et al. [41], as the dose of soluble sodium silicate increases, the absolute value of the Zeta potential in the slurry system will significantly rise, thereby enhancing the electrostatic repulsive double-layer force between particles. At a critical concentration of 1.4 mol/L, this repulsive force dominates the system, making it difficult for the particles to effectively connect and form a continuous spatial network in a static state, and macroscopically, this is manifested as the synchronous drop of the static yield stress τs and the thixotropic loop area.

**Figure 15:**
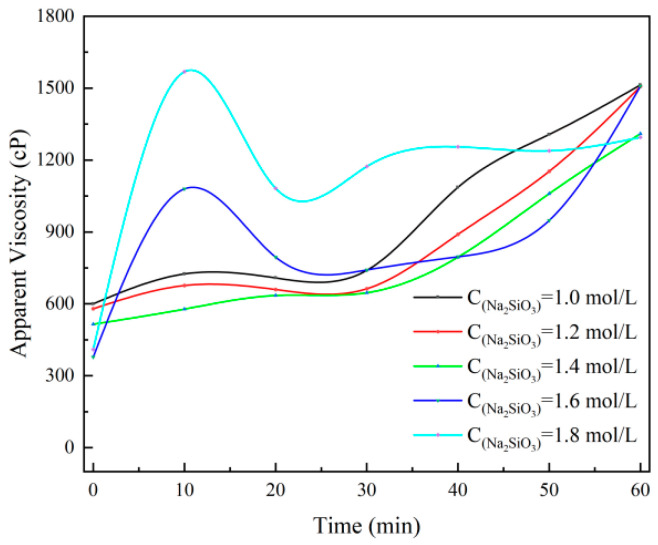
Time-dependent viscosity curves under different alkali activator concentrations.

Figure 15 shows the time-varying viscosity curves at different concentrations of alkali activators. When the concentrations of alkali activators are 1.0, 1.2, and 1.4 mol/L, the time-varying viscosity curves still conform to the same rule. When the concentration of the alkali activator is 1.6 and 1.8 mol/L, the viscosity increases rapidly from 0 to 10 min as the continuous shear proceeds, then decreases rapidly within 10 to 20 min, and steadily increases after 20 min.

At low concentrations, the reaction rate of the system is slow and the structure forms stably. Therefore, the viscosity presents a smooth, monotonically increasing exponential curve. At high concentrations, due to the increase in the absolute value of Zeta potential, the silicate acts as an excellent dispersant [42]. The repulsive force between particles is strong, resulting in a lower initial viscosity measured immediately after the stirring is completed on a macroscopic scale. Within the range of 0 to 10 min, the precursor rapidly dissolves in a strong alkaline environment. The higher chemical activity quickly induces the formation of an early network, thereby causing a sharp increase in viscosity, leading to the first peak. However, as the shear continues, physical damage begins to accumulate, and the structural framework undergoes a viscoelastic shear-thinning behavior. The bound free water is re-released, causing the viscosity to drop within 10–20 min. It is not until 20 min later that the system truly enters the growth stage dominated by stable chemical condensation.

**Figure 16:**
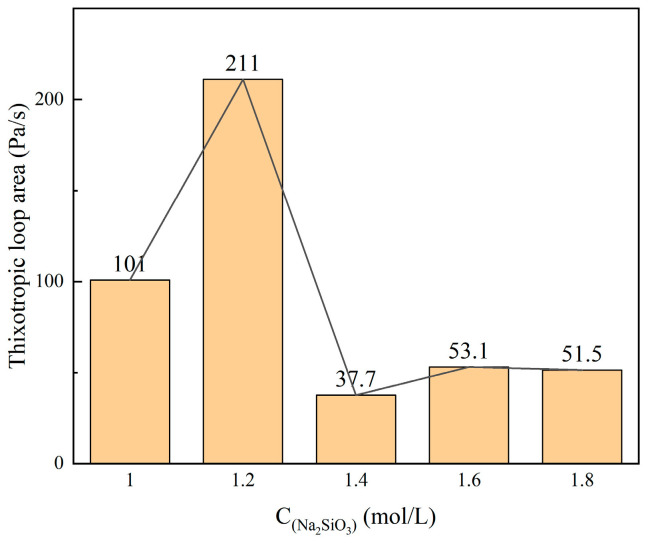
Thixotropic loop area of slurry under different alkali activator concentrations.

Figure 16 shows the area of the thixotropic loop at different concentrations of base activators. When the concentration of the base activator increases from 1.0 mol/L to 1.2 mol/L, the area of the thixotropic loop increases from 101 Pa/s to 211 Pa/s. In this concentration range, the main function of the increase in the concentration of the alkali activator is to enhance the reactivity, facilitate the formation of a faster and denser flocculation network, and increase the area of the thixotropic loop. When the concentration of the base activator increased from 1.2 mol/L to 1.8 mol/L, the area of the thixotropic loop changed from 211 Pa/s → 37.7 Pa/s → 53.1 Pa/s → 51.5 Pa/s. There was a critical concentration in the 1.2–1.4 mol/L range, and the dominant mechanism of the system was switched. Polymeric slurry is essentially a colloidal suspension system. The surface of gel particles carries charges, and ions with opposite charges (counterions) are adsorbed around them to form a double electric layer [43], generating electrostatic repulsion to prevent particle agglomeration. When the concentration of the alkali activator exceeds the critical value, the concentration of counterions in the solution increases [42,44], leading to an increase in electrostatic repulsion between particles. Gel particles tend to exist in a small size and dispersed state, making it difficult to form a penetrating network structure. This finding is consistent with the conclusions drawn by Kashani et al. in their study on the effects of activator type and dosage on the rheological properties of slag pastes. They observed that as the dosage of sodium silicate increases, the absolute value of the zeta potential rises, which enhances the repulsive double-layer forces and promotes the dispersion of particles [41]. As a result, the area of the thixotropic loop drops sharply. This also indicates that the higher the concentration of the alkali activator in the time-varying viscosity curve, the smaller the initial viscosity.

## 4. Conclusions

(1) The rheological type of TSWGGM slurry is mainly determined by the liquid–solid ratio, but it may change under extreme conditions such as low temperature or high concentration of the alkaline activator. Therefore, in actual grouting projects, it is necessary to adaptively select the rheological fitting model based on the specific construction environment and ratio. This is the prerequisite for predicting the migration behavior and pressure evolution characteristics of the slurry.
(2) Increasing the liquid–solid ratio can significantly reduce the flow resistance and ensure long-distance pumping, but excessive physical dilution will widen the particle spacing and severely weaken the viscoelastic structure reconstruction ability of the slurry when it is at rest. It will also increase the risk of slurry loss and ineffective diffusion in the flowing water environment. Therefore, in actual engineering, the liquid–solid ratio must be coordinated and optimized for specific geological boundary conditions.
(3) Higher environmental temperatures and high concentrations of alkaline activators will cause TSWGGM slurry to form a large number of reversible flocculation structures in the early stage of hydration reaction, resulting in pseudo-settlement phenomenon, which seriously hinders the penetration and diffusion of the slurry in the early stage of grouting.
(4) The alkaline activator concentration shows different regulatory effects on the structure formation of fresh slurry in different ranges. At medium and low concentrations, the alkaline activator mainly promotes structure construction by accelerating the depolymerization of precursors and the progress of hydration reaction; while at high concentrations, the electrostatic repulsion force on the particle surface causes the colloidal dispersion effect, which hinders the construction of the spatial web structure, and macroscopically manifests as the synchronous attenuation of static yield stress and viscoelastic structure reconstruction ability.

## Figures and Tables

**Table 1:** Chemical compositions of raw materials (wt%).

Material	Al_2_O_3_	SiO_2_	CaO	Fe_2_O_3_	MnO	SO_3_
Red mud	7.925	22.912	48.922	14.56	0.068	0.498
Fly ash	28.409	48.466	6.34	8.295	0.124	2.865
Slag	15.24	30.54	40.75	0.31	8.58	2.08

**Table 2:** Test plan.

Group	L/S	Temp	M
Activator Concentration (mol/L)	1.4		1.0/1.2/1.4/1.6/1.8
Slurry Temperature (°C)	20	10/20/30/40/50	20
Liquid-to-Solid Ratio	0.5/0.75/1.0/1.25/1.5	0.5	

**Table 3:** Rheological equations for different liquid-to-solid ratios.

Group	Model	Equation	R^2^
L/S = 0.5	$\tau =\tau_{0}+K\gamma^{n}$	$\tau =1.9466+0.4462\gamma^{0.870}$	0.9948
L/S = 0.75	$\tau =\tau_{0}+\mu_{P}\gamma$	τ=0.9014+0.0342γ	0.9474
L/S = 1.0	τ=μγ	τ=0.1538+0.014γ	0.9898
L/S = 1.25	τ=μγ	τ=0.1281+0.0077γ	0.9922
L/S = 1.5	τ=μγ	τ=0.1128+0.0062γ	0.9895

**Table 4:** Time-dependent viscosity equations of slurry under different liquid-to-solid ratios.

Group	Equation	R^2^
L/S = 0.5	$\mu \left(t\right)=441.1e^{0.017t}$	0.9427
L/S = 0.75	$\mu \left(t\right)=73.196e^{0.030t}$	0.9866
L/S = 1.0	$\mu \left(t\right)=51.1432e^{0.044t}$	0.9839
L/S = 1.25	$\mu \left(t\right)=44.0358e^{0.048t}$	0.9835
L/S = 1.5	$\mu \left(t\right)=36.252e^{0.052t}$	0.9858

**Table 5:** Rheological equations under different slurry temperatures.

Group	Model	Equation	R^2^
T = 10 °C	$\tau =\tau_{0}+\mu_{P}\gamma$	τ=1.9841+0.3174γ	0.9983
T = 20 °C	$\tau =\tau_{0}+K\gamma^{n}$	$\tau =1.9466+0.4462\gamma^{0.870}$	0.9948
T = 30 °C	$\tau =\tau_{0}+K\gamma^{n}$	$\tau =5.199\gamma^{0.360}$	0.9425
T = 40 °C	$\tau =\tau_{0}+K\gamma^{n}$	$\tau =3.4656\gamma^{0.516}$	0.9804
T = 50 °C	$\tau =\tau_{0}+K\gamma^{n}$	$\tau =3.0944\gamma^{0.515}$	0.9581

**Table 6:** Rheological equations under different alkali activator concentrations.

Group	Model	Equation	R^2^
C = 1.0 mol/L	$\tau =\tau_{0}+K\gamma^{n}$	$\tau =2.4735+0.6519\gamma^{0.864}$	0.9986
C = 1.2 mol/L	$\tau =\tau_{0}+K\gamma^{n}$	$\tau =2.978+0.6005\gamma^{0.875}$	0.9987
C = 1.4 mol/L	$\tau =\tau_{0}+K\gamma^{n}$	$\tau =1.9466+0.4462\gamma^{0.870}$	0.9946
C = 1.6 mol/L	$\tau =\tau_{0}+K\gamma^{n}$	$\tau =1.9867+0.4307\gamma^{0.962}$	0.9990
C = 1.8 mol/L	$\tau =\tau_{0}+\mu_{P}\gamma$	τ=1.9425+0.5136γ	0.9979

## Data Availability

The original contributions presented in the study are included in the article, further inquiries can be directed to the corresponding author.
